# Gender perspectives of the water, energy, land, and food security nexus in sub-Saharan Africa

**DOI:** 10.3389/fsufs.2022.719913

**Published:** 2022-11-08

**Authors:** Sithabile Hlahla

**Affiliations:** 1 Centre for Transformative Agricultural and Food Systems, School of Agricultural, Earth and Environmental Sciences, College of Agriculture, Engineering, and Science, University of KwaZulu-Natal, Pietermaritzburg, South Africa; 2 Future Water Research Institute, University of Cape Town, Cape Town, South Africa

**Keywords:** poverty alleviation, livelihoods, climate change adaptation, gender-responsive planning, natural resource access

## Abstract

The water, energy, land and food (WELF) nexus has been touted as a cross-sectoral systems approach that presents an opportunity to address the grand challenges related to poverty, unemployment, inequality and climate change, especially in the global South. However, as with any other developmental approach, the WELF nexus needs to mainstream gender, which often lies at the heart of poverty, unemployment, and inequality in sub-Saharan Africa. Access to water, energy, land and food is gendered, and so are livelihood strategies and climate change responses. Inequitable access to these resources, gender inequalities, socio-economic vulnerability and cultural norms contribute to women’s susceptibility to the impacts of climate change and limit their ability to harness opportunities arising from it. Reducing women’s vulnerability to the impacts of climate change in SSA and improving equity in natural resource access and resource use efficiencies will require transformation of gender relations and the active participation of both men and women in decision-making processes. Moreover, policies and interventions that cater to the WELF nexus need be updated to be more gender-aware and sensitive, as this will also contribute to addressing Sustainable Development Goal 5, in addition to Goals 1, 2, 6, 7, and 15.

## Introduction

Water, energy, and land are important for many life supporting functions and the provision of basic human needs (German Development Institute, 2013). These resources are crucial contributors to food security and development (German Development Institute, 2013; Ringler et al., 2013). Due to growing natural resource scarcity, the inter-linkages that exist between these sectors have become more pronounced, as evidenced by growing trade-offs and the search for cross-sector efficiencies (Ringler et al., 2013). Access to these resources and their sustainable management are the basis for inclusive sustainable development, and poverty reduction (German Development Institute, 2013). However, resource access for the poor in sub-Saharan Africa remains both a practical and policy challenge, and inequitable access to basic resources is especially prevalent among women (Villamor et al., 2018). Women’s access to water, energy, land, and food resources in sub-Saharan Africa is further explored in this chapter.

The interdependencies that exist between water, energy, land and food are numerous and multidimensional, and these interlinkages are referred to as the water-energy-land-food (WELF) nexus (Ringler et al., 2013; OECD, 2018; GWP SA, 2019). These resources are crucial to human existence and how they are governed affects outcomes in terms of social equity, externalities, and socio-ecological resilience (Albrecht et al., 2018). Moreover, sector policies for these resources are intertwined, in particular, in their trade-offs (German Development Institute, 2013). Policies for one sector often bear consequences or negative externalities for the other three sectors, on local, national, regional or global scales (German Development Institute, 2013; Serdeczny et al., 2017; OECD, 2018). These interlinkages add to current pressures on water and land as well as on resources that fuel the energy system, exacerbating existing scarcity problems, as the demand for food, water and energy is expected to increase by 30–40 percent by 2030 (German Development Institute, 2013). Therefore, the implementation of the nexus requires policies, institutional arrangements and procedures that can take these trade-offs and synergies into account (German Development Institute, 2013). Failure to do so will generate high costs to the economies of SSA and exacerbate inequalities across countries and social groups, both now and in the future (OECD, 2018).

Water plays a crucial role in food and energy production [especially hydro-energy production which is a major source of energy in southern Africa (Nhamo et al., 2018), and in sustaining the ecosystems that support agriculture and other economic activities that are important for achieving food security (GWP SA, 2019)]. Energy is necessary for food production and for water supply (including the extraction, purification, and distribution of water) (Nhamo et al., 2018; GWP SA, 2019). Interactions among these nexus components play a vital role in the living standard outcomes of marginalized households, and can be captured and/or affected through changes in household behaviors or activities (Villamor et al., 2018). Access to, control over, and the use of WELF resources are also influenced by gender, however, not many studies have taken this into consideration nor have they addressed the potential for differential effects of WELF nexus interventions with respect to gender (Villamor et al., 2018; Khadka, 2022; Sani and Scholz, 2022). Women, who constitute two-thirds of the world’s poor, are key stakeholders in nexus sectors, however, they face significant structural barriers (Khadka, 2022; Sani and Scholz, 2022). Moreover, the working culture in these sectors is still guided or influenced by masculine attitudes, behaviors, and mindsets and women struggle to access these men-dominated spaces (Khadka, 2022). Very little effort is made to understand and tackle systemic gender issues related to water, energy, land, and food within the nexus sectors (Khadka, 2022). This is evidenced by the fact that global discussions on the WELF nexus have under-represented the linkages that exist between gender and the nexus (Villamor et al., 2018; Purwanto et al., 2021). The linkages refer to women’s access to, and their role in the utilization of these resources. For example, women are responsible for providing food for their households in addition to fetching fuelwood for cooking and potable water, which are unpaid productive activities (Villamor et al., 2018). Moreover, the majority of agriculturalists in SSA are women (Ruiters and Wildschutt, 2010; Villamor et al., 2018), and easy and equitable access to these resources will promote women’s socio-economic development while contributing toward gender equality. Therefore, gender is relevant to the WELF nexus agenda and should be taken into account, especially at the local level. Climate change exacerbates insecurities of WELF resources, while changes in the availability of these resources, access, and security, shapes the manner in which individuals, communities and countries respond to the changing climate (Sultana, 2018). Social differences such as gender and class impact the ways in which the impacts of climate variability and change are experienced and responded to (Sultana, 2018). Against this backdrop, this article seeks to explore the interlinkages that exist between the WELF nexus and gender and how these interlinkages can facilitate greater and more effective climate change response strategies and resource use efficiencies in sub-Saharan Africa. Policies and interventions that cater to the WELF nexus need be updated to be more gender-aware and sensitive. This will also contribute to addressing Sustainable Development Goals 1 (no poverty), 2 (zero hunger), 6 (clean water and sanitation), 7 (affordable and clean energy), and 15 (life on land), in addition to Goal 5 (gender equality).

## Research methods

The study used secondary data to review literature on gender and the water, energy, land, and food security nexus in sub-Saharan Africa. The literature focussed on six main themes, which became the search terms. These themes were:

- Gender and water in sub-Saharan Africa
- Gender and energy in sub-Saharan Africa
- Gender and land in sub-Saharan Africa
- Gender and food in sub-Saharan Africa
- Gender and climate change in sub-Saharan Africa
- Climate change and WELF resources.

The literature published in academic and non-academic sources was assessed, including journal articles, book chapters and gray literature. The databases used for the search were Google and Google Scholar. The findings were then reported under the various themes.

The majority of literature found in the study focused solely on women, despite a focus on gender. A similar finding was found by Pouramin et al. (2020) who conducted a systematic review on gender and water. The authors reported that nearly half the studies (46%) focused exclusively on the experience of women and girls and did not consider impacts on men.

## Gender and WELF nexus resources in sub-Saharan Africa

### Gender and water

Access to improved water and sanitation are important human rights, however, 780 million people lack access to safe water and one third are experiencing some form of economic or physical water scarcity (IFAD, 2012; German Development Institute, 2013; Armah et al., 2018). Access to improved water sources has increased over the last 30 years in the SSA, however, this access is gradually being eroded due to the region’s high rates of population growth and urbanization (Dominguez et al., 2012; Ahmed, 2018; Armah et al., 2018). The high population growth and urbanization rates have not been accompanied by economic growth or investment in housing, water, and sanitation infrastructure (Armah et al., 2018). Furthermore, climate change is compromising the provision of water supply services in the region resulting in more frequent droughts and water shortages (Dominguez et al., 2012). Amidst these challenges, there is also growing competition for water within different sectors, making it increasingly difficult for people, especially marginalized women, who are the primary users, providers and managers of water in their households, to access the resource for productive, consumptive and social purposes (WSP, 2010; IFAD, 2012; Jong et al., 2013). The sectors competing for water include industry, agriculture, power generation, domestic use, and the environment (IFAD, 2012; Jong et al., 2013).

Women and men have differential roles, rights and responsibilities with regards to water (Ahmed, 2018). Women face inequity in access to water resources, despite the fact that they are primarily responsible for the management of household water supply, sanitation and health (Allély et al., 2000; Armah et al., 2018; Pouramin et al., 2020; UN, 2018). For example, in schools in Malawi and Ethiopia, girls lack access to adequate sanitation and hygiene in the form of clean water supplies and sufficient latrines (Fleifel et al., 2019). Moreover, women and girls in rural areas are forced to walk long distances to fetch water, preventing them from undertaking other activities or participating in education (Palacios-Lopez et al., 2017; Ahmed, 2018; Pouramin et al., 2020). For example, in Mauritania, Somalia, and Tunisia, an average distance of a trip to collect water is 4-5 kms and takes 33 mins each way (Connell, 2017). The physical security of the women and girls is also threatened during their walk as they generally travel long distances, on unsecured paths, alone, leaving them vulnerable to sexual violence and attacks (Fleifel et al., 2019). It is estimated that more than two-thirds of the SSA population leave their homes to collect water, and the majority of rural water systems are often non-functional, making water collection even more difficult (Palacios-Lopez et al., 2017).

Access to water is invariably linked to sanitation and hygiene. Therefore, inequitable access to water can lead to poor sanitation and health burdens for women (WaterAid Canada, 2017; Pouramin et al., 2020). Given their traditional role as water purveyors, sexual and reproductive health needs, and their role as caretakers within the household, access to clean water and sanitation is of great importance to women and girls (WaterAid Canada, 2017). If the water is contaminated with infectious microorganisms, the risk of contracting waterborne diseases such as cholera is higher, and women are at higher risk of contracting and transmitting this disease due to their water provisioning role (Pouramin et al., 2020). Similarly, women can contract urinary tract infections (UTIs) due to poor menstrual health management which is often due to the failure to access the additional resources required for good menstrual health (Pouramin et al., 2020). This can lead to girls missing school and overall reduced education (Pouramin et al., 2020), or women missing out on income generating opportunities (WaterAid Canada, 2017). Carrying buckets of water for protracted periods of time can also lead to fatigue and back and arm injuries. As transporting water takes up considerable time and energy, it places high demands on the metabolism, and can result in pressure on the skeletal system leading to early arthritis (Palacios-Lopez et al., 2017). Therefore, water transporters are vulnerable to musculoskeletal damage and early degenerative bone and soft tissue damage which may compromise their ability to access water in the long-run (Palacios-Lopez et al., 2017). The provision of water sources in proximity to households would make water collection more efficient and improve domestic and personal hygiene (Palacios-Lopez et al., 2017). Some authors have also found that hygiene practices and hygiene-related health outcomes are directly related to how far the water source is from a household (Palacios-Lopez et al., 2017). People who are denied access to improved water and sanitation services face “diminished opportunities to realize their potential” (Armah et al., 2018, p. 2).

Securing water and sustained access to water are important for achieving food security and improving the rural livelihoods in SSA. Women play a fundamental role in food security through their knowledge of crop production, local biodiversity, soils and local water resources (Jong et al., 2013). Women make substantial contributions to the rural economy of SSA as farmers, laborers and entrepreneurs, comprising at least 40% of the agricultural labor force in the region (Palacios-Lopez et al., 2017). However, they are often excluded from decision-making processes regarding water and new agricultural water management systems and other projects and initiatives concerning the allocation of natural resources (Jong et al., 2013). Excluding women from decision-making processes results in resources being less accessible to them and important issues such as menstrual hygiene management, are viewed as niche issues and taboo (WaterAid Canada, 2017). The women also lose out on opportunities to generate income, for example, agricultural activities which depend on the availability and accessibility of water (WaterAid Canada, 2017). It is important for both men and women to participate in decision-making in order to determine what they need, if they can make any contributions and in what capacity (Jong et al., 2013). Gathering information on the experiences and knowledge of both men and women will allow for a better understanding of existing practices and challenges, and help to identify the problems that need to addressed first (Jong et al., 2013). This will also lead to better investment decisions (Jong et al., 2013). The involvement of both men and women will also help to identify any conflicts that might exist between different socio-economic and ethnic groups and ways can be found to prevent or solve them (Jong et al., 2013).

Securing women’s access to water is crucial to tackling two goals of sustainable development, i.e., Sustainable Development Goals (SDGs) 5 (gender equality) and 6 (clean water and sanitation). The water and sanitation sector has the potential to contribute to redressing inequality and can greatly improve the social, political and economic position of women (WSP, 2010). Providing women with sufficient access to improved water sources tackles time poverty by freeing up the women’s time to allow them to participate in social, economic, and political activities, and decision-making (WSP, 2010; Ahmed, 2018). Armah et al. (2018) assert that compositional factors such as the household head’s sex, age, and level of education, and the size of the household contribute significantly to the disparities in access to improved water sources and sanitation facilities in SSA (Armah et al., 2018). Female-headed households in SSA are more likely to advocate for access to improved water sources and sanitation due to their roles within the household, and are likely to pay more attention to such issues than men (Armah et al., 2018). Therefore, it is important that gender is mainstreamed into any programme, policy, initiative or discussions about water. Gender mainstreaming is strategy to promote gender equality and women empowerment at all levels of development by ensuring that gender perspectives are central to all activities (UN Women, 2014). These activities include policy development, research, advocacy/ dialogue, legislation, resource allocation, and planning, implementation and monitoring of programmes and projects equality (UN Women, 2014). There is also need for capacity building and bottom-up approaches, which can also help to build social capital and support autonomous adaptation efforts (Ahmed, 2018). In addition, in order to tackle the impacts climate change will have on water provision, flexible, adaptable, and gender-conscious solutions are required as well as the design and development of water supply infrastructure which can withstand the changes and reduce the vulnerability of systems (Dominguez et al., 2012).

### Gender and energy

The energy sector is a key driver behind a number of critical environmental pressures, including anthropogenic greenhouse gas emissions (GHG) and land and water use and degradation (German Development Institute, 2013). It is estimated that energy-related emissions (including transportation, electricity and heat, buildings, manufacturing and construction, fugitive emissions and other fuel combustion) account for 73% of the global total GHG emissions (Ge and Friedrich, 2020). However, the sector is also undergoing tremendous external stress and changes due to several factors including climate change and the COVID-19 pandemic which has slowed down efforts to increase electrification rates as governments are prioritizing immediate public health concerns and economic crises (IEA, 2020). The energy sector in SSA is underdeveloped, making access to energy one of the region’s greatest obstacles to social and economic development, at a time when the increasing populations and prospects of economic growth require more energy (Hafner et al., 2018). Energy use per capita in SSA is equivalent to one-third of the world’s average and 25% of that of the Middle East and North Africa (Hafner et al., 2018). South Africa is the only country in the region whose per capita energy use exceeds the world average (Hafner et al., 2018). Across SSA, there are large disparities in per capita consumption between urban and rural areas, with cities enjoying better access to modern forms of energy (Hafner et al., 2018).

Energy poverty is one of the most critical development challenges in SSA as many people face inadequate and unreliable access to modern energy services and rely heavily on traditional biomass fuels such as wood, charcoal, dung, and agricultural wastes for cooking and heating (Lambrou and Grazia, 2006; Danielsen, 2012; Hafner et al., 2018). Energy poverty also has a strong gender element (OECD, 2021). It is estimated that 30% of households live in energy poverty, and 81% of SSA depends on woody biomass energy especially for cooking, household and economic activities (World Bank, 2011; Bildirici and Özaksoy, 2016; OECD, 2021). Unfortunately, the inefficient use of solid biomass fuel for cooking and lighting causes indoor air pollution which kills ~600,000 people per year (Alstone et al., 2011; Hafner et al., 2018; Banerjee, 2019). Women and young children are the most affected by this pollution because they spend more time inside the house and next to the stove while food is being cooked (Hafner et al., 2018).

The majority of people who have no access to basic modern energy services reside in the rural areas of SSA. Such deprivation often has negative impacts on economic and social development, health, time education and fulfillment, and gender equality (Lambrou and Grazia, 2006; Danielsen, 2012; Hafner et al., 2018). Women and men play different gender-defined roles in energy production, distribution and utilization in households, communities and the market (UNDP, 2012), and women experience energy poverty differently and more severely than men (Alstone et al., 2011; Danielsen, 2012). However, energy policies in the region are gender blind (Johnson et al., 2019). People with no or limited access to energy tend to be generally poorer than those with energy access; they are less productive; face heavier work; are more exposed to health risks; and lack the benefit of modern technologies and communication (Hafner et al., 2018). For example, in rural areas, energy is the primary responsibility of women and they spend most of their days engaged in time-consuming and physically draining subsistence activities which include the fetching of water and biomass fuels (Lambrou and Grazia, 2006; Alstone et al., 2011; Danielsen, 2012; UNDP, 2012; Hafner et al., 2018; Johnson et al., 2019). This is time they should spend in school or on earning additional income or improving their labor productivity which can empower them and promote socio-economic development (EEP S&EA, 2017; Hafner et al., 2018). Unfortunately, due to a lack of information, awareness, and tangible action plans for development institutions and the private sector, the role of women in the energy sector is often overlooked in developing countries or misinterpreted (Alstone et al., 2011).

Access to energy is a human right, and gender impacts this right in two ways: supply (production) and consumption (Banerjee, 2019; Johnson et al., 2019). On the consumption side, women have major energy-consuming responsibilities in and around the household and community, while on the supply side, women in the rural areas of SSA, as mentioned earlier, play a major role in the production and procurement of energy, particularly, traditional fuels such as firewood and other woody biomass (Johnson et al., 2019). However, it is important that women and men have different energy requirements (Banerjee, 2019). For example, women may opt to place illumination devices in kitchen, while men may opt to place them in living room to facilitate social gatherings (Banerjee, 2019). Energy access tends to be determined by intra-household decision-making, the social position of women, and the value attached to women’s labor (Danielsen, 2012; Banerjee, 2019). There is no economic value associated with biomass collection, therefore, there is a lack of recognition of this type of women’s work, and the other multiple roles they fulfill in the household (Danielsen, 2012). Moreover, women’s work in agriculture and as entrepreneurs has received limited recognition, while investments into improved cooking technology has not been prioritized at both the household and national levels (Danielsen, 2012). Studies have shown that access to electricity within the household has indirect but strong impact on women (Banerjee, 2019). For example, household electrification increased female employment and school attendance in rural areas, especially amongst teenage women (Banerjee, 2019), while in rural health centers, access to electricity has been instrumental in much safer child deliveries (Banerjee, 2019). Other reasons for the rights failures with regards to women’s rights to energy is the fact many women lack control over land and property, which affects their ability to benefit equally to men from energy facilities, including, solar systems, wind turbines, and bio-fuel plantations, which require land (Danielsen, 2012). A study on access to electricity for business based in Ethiopia, Ghana, Kenya, Tanzania, and Zambia revealed that electricity connections for women-headed businesses are generally delayed when compared to their male counterpart (Banerjee, 2019). This discrepancy has major ramifications for self-employed women, and some of these women have to resort to paying bribes secure electricity connections (Banerjee, 2019). A lack of income also acts as a barrier for women to invest in technology that can significantly improve the productivity of their labor (Danielsen, 2012). Women lack access to credit which affects their ability to pay the up-front costs of improved energy technology or connection fees to the electricity grid (Danielsen, 2012). They also have limited access to extension services and education, limiting their abilities to become energy entrepreneurs and earn an income (Danielsen, 2012). Lastly, women internalize social norms that place a low value on their worth and contribution (Danielsen, 2012). This likely has a negative impact on their access to modern energy services, as well (Danielsen, 2012). Unequal gender and power relations can hinder women’s ability to participate and voice their energy needs in decision-making at all levels of the energy system (Danielsen, 2012).

Energy is key to development and poverty alleviation (UNDP, 2012), and decision-making about its use has complex linkages with policies pertaining to poverty, food security, health, population, gender disparities, environmental quality, investments, foreign exchange, trade and national security (Lambrou and Grazia, 2006). However, despite a growing knowledge base on the linkages between gender, energy and poverty, international efforts to promote the energy rights of women are still inadequate, and the gender and rights failures are being replicated (Lambrou and Grazia, 2006; Danielsen, 2012). Gender equality tends to be viewed as a predominantly political issue, which is not related to technical concerns about energy production and supply (Lambrou and Grazia, 2006). Therefore, energy planners and policy-makers seldom consider women, and concerns about women are treated as ‘added on’ factors which are not directly relevant to energy issues (Lambrou and Grazia, 2006, p. 7). Moreover, the energy sector mostly employs men, with women mainly being considered as the beneficiary or customer (EEP S&EA, 2017). The rights failures within the energy system are reflective of the governance malfunctions at all levels (Danielsen, 2012). Therefore, in order to overcome the challenges associated inequitable access to energy, the energy system governance, policy and programmes need to address structural gender inequalities at the level of institutions (Danielsen, 2012).

It is necessary to mainstream gender in energy-related policies, programmes and initiatives at all levels of government (Lambrou and Grazia, 2006), and engage more women as grassroots workers in the electrification programmes (Banerjee, 2019). Policies need to illustrate that gender equality is a priority, while energy programmes and interventions should be designed to create opportunities for women’s empowerment, and gender equality (Lambrou and Grazia, 2006). Women should be encouraged to participate in energy-related decision-making at the national, local and household levels, and at an organizational level, space and opportunities should be made available to both women and men (Lambrou and Grazia, 2006; Banerjee, 2019). Countries such as Zimbabwe and Rwanda are already working toward the increased representation of women in the energy sector (Banerjee, 2019). The Ministry of Energy and Power Development in Zimbabwe has identified a gender focal point to coordinate the implementation of gender mainstreaming in the energy sector, while the government in Rwanda, encourages women’s participation in the planning, design and execution of energy programmes (Banerjee, 2019). As women are not a homogeneous group, it is also necessary to consider gender disparities within the particular social, economic and political contexts (Lambrou and Grazia, 2006). Factors such as culture, income, social class, religion, family status and geographical location need to be taken into account in energy decision-making (Lambrou and Grazia, 2006).

Efforts to mainstreaming gender into energy approaches need to go beyond a focus on technological changes such as replacing candles and traditional cookstoves with cleaner and more efficient alternatives, or meeting the immediate or practical needs of women that affect their daily lives and practices (Johnson et al., 2019). It is also necessary to address the broader socio-cultural empowerment or strategic’ needs required to transform gender divisions of labor, power, and control, need to be addressed (Johnson et al., 2019). Adopting people-centered, gender-differentiated approaches to energy planning guarantees that women and men have equal opportunities to access, participate, and benefit from en-ergy sector initiatives (EEP S&EA, 2017). This will also help to tackle two goals of sustainable development, i.e., Sustainable Development Goals (SDGs) 5 (gender equality) and 7 (affordable and clean energy). This requires the development and implementation of initiatives that integrate gender equality into affordable clean energy programmes and ensuring that the benefits of affordable clean energy also contribute to gender equality (EEP S&EA, 2017). The participation of women in decision-making, and the design, distribution, management, and production of sustainable energy solutions improves development outcomes (Johnson et al., 2019), and.is essential to the realization of the goals of sustainable development goals (EEP S&EA, 2017). Gender-sensitive energy programmes can also ease the double burden of lack of sufficient energy and poverty that women, especially rural women, face as they perform traditional household and community roles (Lambrou and Grazia, 2006). Meeting the energy needs of women can lead to improved health and wellbeing of entire communities; provide them with opportunities for education and income generation, thereby improving their social and economic statuses, while improving the standard of living for themselves and their families and communities, and greater security (Lambrou and Grazia, 2006; Johnson et al., 2019). Capacity development and the establishment of a monitoring system to assess the progress toward eliminating energy poverty and gender mainstreaming is necessary as this can improve women’s access to energy and improve accountability to women’s energy rights (Danielsen, 2012). Access to affordable, sustainable and clean energy is necessary for gender equality and wellbeing (OECD, 2021).

### Gender and land

Land is a productive asset and an immovable factor of development which serves as a major source of wealth for the poor, and plays a key role in social relationships (Akinola, 2018; Chigbu, 2022a; Murphy and Fogelman, 2022). In sub-Saharan Africa, the issues women face regarding equal access to, ownership of, and control over land are complex (IIED, 2015), despite the fact that, after the 1990s, land reforms in countries in the region started incorporating gender aspects in legal provisions to protect women’s land rights (Ghebru, 2019). This complexity is attributed to the fact that individual property rights co-exist with customary laws and the majority of agricultural land is not registered and does not have formal ownership documents (Slavchevska et al., 2020). In rural areas, customary tenure regimes, which are driven by lineage or clan control, shape people’s access and ownership of land (Goldstein et al., 2016; Akinola, 2018; Massay, 2019). These laws tend to favor men, therefore, women, are less likely than men to own land, even when they make a significant contribution to agricultural labor (Jong et al., 2013; Goldstein et al., 2016; Massay, 2019), and where they do own land, the plots are usually smaller than those owned by men and are often of poorer quality (FAO, 2011; IIED, 2015). Women can likely gain “secondary” land use rights, which are undocumented, through a male spouse or relative, however, in the event of divorce or the spouse’s death, they can lose these rights (Goldstein et al., 2016; IFAD, 2013). Widows are the most likely to lose their land and other assets, further disempowering them and their children (Akinola, 2018). The lack of tenure security means that women, in particular, widows and female-headed households, are amongst the most vulnerable in society (IFAD, 2013). Given the role of women in household subsistence production and welfare, steps need to be taken to strengthen their rights to land, thereby contributing toward gender equality and poverty reduction (IFAD, 2013), Sustainable Development Goals (SDGs) 5 and 1, respectively.

There are several female-headed households in SSA due to the death of husbands, high rates of divorce, and the prevalence of ‘single motherhood’ in countries such as South Africa. These households are in need of land resources as economic and productive assets (Akinola, 2018). Secure access to, and ownership of land are necessary for the improvement of women’s socio-economic status, well-being, and self-esteem (Akinola, 2018; Massay, 2019; Slavchevska et al., 2020; IFAD, 2013). Studies have revealed that gender-equitable division of land has numerous benefits, including (i) increased rural productivity, (ii) increased bargaining power within the household, (iii) reduced domestic violence, (iv) improved child nutrition and health, and (v) improvements in household welfare (IIED, 2015; Massay, 2019; Slavchevska et al., 2020). Women tend to spend a larger portion of household income on food, therefore, any increase in female landholdings will lead to an increase in the amount of income spent on food and child education (Massay, 2019), which will have positive impacts on the household. Massay (2019) reports that the children of women who own land are less likely to be severely underweight because the women oversee household decisions. As a result of these benefits, some countries in the region have started incorporating gender-equitable land governance into their national legal frameworks (IIED, 2015). For example, in Senegal, the national law states that “men and women have equal right to access and ownership of land” (IIED, 2015, p. 3), and in Ghana, the Land Act 2020 (Act 1036) prohibits discriminatory practices based on gender, and calls for gender considerations to be taken into account when staffing the Customary Land Secretariat (Republic of Ghana, 2020). In Kenya, the Constitution advocates for the “elimination of gender discrimination in law, customs and practices related to land and property in land” (IIED, 2015, p. 3), however, implementation of the law has not yielded much success with respect to women’s ownership of land (Akinola, 2018). Chigbu (2022b) notes that while policy articulation by the government on the rights of women farmers has shifted, and progressive changes are being made, we are far from a deep change and exclusionist attitudes to making decisions still exist. Securing women’s land rights is a complex process, especially where sociocultural patterns, structural and economic impediments, and power imbalances hinder efforts (IIED, 2015; Akinola, 2018). Some households may overlook national laws regarding gender equality and uphold their traditional practices which greatly disadvantage women. Moreover, many women in SSA have limited access to credit facilities and lack the financial means to purchase land (Akinola, 2018).

Any efforts to secure land rights for women and tackle gender inequalities in land governance need to consider local contexts and gender dynamics, and move beyond generalizations about customary practices and seek opportunities promote women’s voices and decision-making power (IIED, 2015; Massay, 2019; Chigbu, 2022b). Within these households or families, the person who decides on land management and participates in community meetings has the decision-making power (IIED, 2015). Unfortunately, women are seldom involved in decision-making processes concerning land. While gender inequalities around land governance are predominantly linked to women’s capacity to hold and inherit tenure rights, they are also a result of women’s lack of participation in decision-making processes concerning land and gender discrimination in sociocultural and political relations (IIED, 2015; Massay, 2019). Moreover, many national and international discussions about land, including commercial pressures and ‘land grabbing’, are gender blind and have very little input from or representation of women, in particular, rural women (IIED, 2015). The focus of some of these discussions is on the needs of rural women in terms of equal access to and control over land, however, the voices and opinions of these women are often not included (IIED, 2015). Exclusionist land governance systems cannot achieve the expected development dividends that would enable the fulfillment of the global development agendas (Chigbu, 2022b). Therefore, these discussions would benefit from the participation and involvement of rural women as they can provide an understanding of their specific needs around access to land and the main drivers behind these needs (IIED, 2015). Steps need to be taken to ensure the voices of women are heard. Time should also be taken to understand the context and identify some of the entry points women can use to claim their rights. These same entry points can then be utilized to support women’s empowerment through exercising agency, while claiming and realizing their rights (Massay, 2019). Women’s secondary rights to land also need to be recognized by governments as being equal to men’s rights, spousal rights need to co-registered, and women’s inheritance rights need to be recognized (IFAD, 2013). Furthermore, the process of land registration for women needs to be improved as Akinola (2018) states that some women in both urban and rural areas who have been granted access to land have struggled to obtain title deeds, while registration for such lands tends to be expensive for marginalized groups, and is very difficult. In countries such as Ethiopia, Ghana, Madagascar, land rights registration is subject to high costs and bribes (Ghebru, 2019). Unfortunately, women face similar challenges if they litigate or appeal land disputes (Ghebru, 2019). Ghebru (2019) asserts that in order for any formal land registration reform to be considered as gender-sensitive, the formal costs and fees associated with such reforms should be affordable, while informal costs, such as bribes, should be eliminated or discouraged. “Whether a governance system can deliver pro-poor and gender-balanced outcomes depends on its structure. It also depends on whether all the stakeholders involved in the gender continuum play their respective roles in land governance” (Chigbu, 2022a; p. 5).

Women from rural areas have launched activism campaigns to fight against rural gender discrimination related to land rights (Massay, 2019). For example, civil society organizations (CSOs) such as Kenya Land Alliance, Rwanda Land Alliance, Uganda Land Alliance, the National Land Forum in Tanzania, National Land Committee in South Africa, and the Namibian NGO Federation are advocating for the land rights of women, pastoralists, the landless and other marginalized people in their countries (Massay, 2019). It is important to note that women in urban areas (including townships, informal settlements or slums) tend to have more property rights than their rural counterparts (Akinola, 2018). This is largely because of the erosion of cultures and traditional norms in urban areas; the higher level of education; and the monitoring of legal provisions by government institutions (Akinola, 2018). For example, in Ghana, women in urban areas own 23% of couple’s wealth, as opposed to 15% owned by rural women (Akinola, 2018).

Land tenure security is important for the socio-economic development of women in SSA. Securing land rights for women is also important for addressing agricultural production, and climate change mitigation and adaptation (Massay, 2019), as this will motivate them to utilize the land in a sustainable manner, while contributing to conservation efforts and livelihood strategies that are adapted to the changing climate, without fear of losing their land (Gioverelli and Scalise, 2016; Veit, 2019). However, the right to land is still denied to many women and this has resulted in endemic land underutilisation and low agricultural productivity (Akinola, 2018). In addition to drafting of laws to allow for gender equality with regards to land rights, there is need to change traditional belief systems in SSA (Akinola, 2018). Changing traditional belief systems can be achieved through education and awareness raising, especially among the youth. The most complex constraint to the achievement of land and property rights for women is the cultural one (Akinola, 2018). Men need to be receptive to the change in gender relations and they need to view women as partners (Akinola, 2018). Strengthening women’s land tenure security will contribute to human rights and at least six SDGs: goals 1 (no poverty); 2 (zero hunger); 5 (gender equality and empowering women and girls); 11 (sustainable cities and communities); 15 (life on land); and 16 (peace and justice and strong institutions). Chigbu and Enemark (2022) concur, stating that sustainable development cannot be achieved without ensuring that women, globally, have equal rights and opportunities to access and enjoy the benefits of land resources. Achieving gender objectives (e.g. gender equity and gender equality in natural resource use and land tenure) by 2030 requires action to eliminate the root causes of discrimination that still prevent women’s access to land and the enjoyment of land rights Chigbu and Enemark, 2022). Making gender vision of development will be largely determined by how land and natural resources are governed at the three levels of government– national, sub-national and local levels (Chigbu and Enemark, 2022). Non-governmental and civil society organizations play an important role in advocating for women’s land rights and they will continue to be crucial in the fight for gender equality and in ensuring that women’s voices are heard.

### Gender and food

Women play an important role in food production, food distribution, and food utilization (Habtezion Z., 2012). They also participate in a range of community-level activities that support agricultural development, for example, soil and water conservation, afforestation and crop domestication (Habtezion Z., 2012). Traditionally, within the household, women play a critical role in securing food for their families (Hyder et al., 2005). They tend to shop for food and/or grow it in their gardens (Ben-Ari, 2014). Women produce up to 80% of food for household consumption and sale in local markets (Ben-Ari, 2014). Agriculture is central to women’s livelihoods, however, they encounter several barriers in accessing productive resources, assets, services, and markets (FAO, 2011; Habtezion Z., 2012; Jong et al., 2013; Sakho-Jimbira and Hathie, 2020). They tend to work on smaller farms, have access to fewer livestock and have a greater overall workload which includes low-productivity activities such as collecting water and fuelwood (FAO, 2011; Jong et al., 2013). In addition, women have less access to education, agricultural information and extension services, technology, credit and other financial services (Hyder et al., 2005; Jong et al., 2013). They are also less likely to use modern technological inputs such as improved seeds, fertilizers, pest control measures, irrigation, time-saving equipment, and mechanical tools (FAO, 2011; Meinzen-Dick et al., 2012; Rodgers and Akram-Lodhi, 2019). These barriers make it difficult to participate effectively in agricultural production, increasing their vulnerability to food and nutrition insecurity (Habtezion Z., 2012), and negatively impacting their income generation opportunities (Sakho-Jimbira and Hathie, 2020). This is one of the major reasons why the agriculture sector is underperforming in SSA-women do not have access to the resources and opportunities they need to “make the most productive use of their time” (FAO, 2011, p. 3; Bjornlund et al., 2020). Furthermore, they lack a voice in management decisions-decisions affecting their lifestyles and livelihoods (Hyder et al., 2005; Ben-Ari, 2014).

Sixty percent of the population in SSA population are smallholder farmers (Sakho-Jimbira and Hathie, 2020), and women make up at least 40% of the agricultural labor force (Palacios-Lopez et al., 2017). However, the contribution of women to agriculture in SSA is often not formally recognized and they face obstacles to engaging on equitable and fair terms (Villamor et al., 2020). The productivity and success of women in agriculture hinges on the availability of land and water-resources in which their access is limited. This limited access means that they achieve lower yields even though they are equally good at farming as men (FAO, 2011; Meinzen-Dick et al., 2012). Studies have shown that if women farmers had the same level of access to resources as men, they can achieve the same yield levels (FAO, 2011). It is estimated that the yield gap between men and women ranges between 20 and 30 percent and the gap is largely due to differences in resource use (FAO, 2011). Transforming gender relations in agriculture by allowing women to have the same access to resources as men and empowering them as decision-makers will have multiplier effects on production, productivity, efficiency, inclusive growth and poverty reduction in the region (Farnworth et al., 2013; Ben-Ari, 2014; Sakho-Jimbira and Hathie, 2020). Rodgers and Akram-Lodhi (2019) concur, stating that closing the gender gap in SSA has the potential to increase crop production by up to 19%, boost agriculture and overall GDP, while lifting thousands of people out of poverty.

Developing and implementing gender-sensitive agricultural and nutrition policies can bring attention to women as key actors of food and nutrition security, while addressing the challenge of feeding an increasing population (Sakho-Jimbira and Hathie, 2020). The transformation should include gender-responsive budgeting, which will ensure that commitments to women’s equality are translated into measures to help finance equity measures in the agricultural sector (Farnworth et al., 2013). In addition to increasing women’s agricultural productivity, it is necessary to invest in women’s entrepreneurship and technical skills, their participation in niche markets, and higher value-added activities (Sakho-Jimbira and Hathie, 2020). It will also be beneficial to explore the complex interactions that exist between gender and agriculture within the sub-Saharan African farming systems in order to have a better understanding of them (Meinzen-Dick et al., 2012). One way this can be achieved is through investing in women’s involvement in agricultural research (Sakho-Jimbira and Hathie, 2020). This will allow for perspectives and insights that are more gender-sensitive which can assist to overcome the challenges female farmers are facing on the ground (Sakho-Jimbira and Hathie, 2020). The number of women undertaking in agricultural research in SSA has so far increased by 24%, from <9,000 in the year 2000 to more than 15,000 in 2014 (Sakho-Jimbira and Hathie, 2020). This is likely due to increased access to education for girls, which has resulted in more women enrolling into agricultural sciences, and sciences (MacNeil, 2017).

## WELF nexus, gender, and climate change: Interlinkages

Water, energy, land and food resources are important to the livelihoods of women, however, a gender gap exists in all sectors which limits their productivity and their ability to contribute toward wider social and economic development goals (FAO, 2011; Njuki, 2021). Moreover, gender is missing from the WELF nexus frameworks (Villamor et al., 2020). Attempts by the individual sectors to address the gap have not been successful due to numerous factors including political will, budgetary constraints, and the unwillingness of men to change their mindsets (Thuo et al., 2017). Moreover, resource management and allocation in sub-Saharan Africa is conducted using a silo approach which is contributing to the region’s failure to meet its development targets, exacerbating its vulnerabilities, and leading to inequitable access to resources (Nhamo et al., 2018). This lack of coordination of WELF nexus synergies and trade-offs in planning is compromising gender equality and the sustainability of development initiatives (Nhamo et al., 2018).

The security of water, energy, land and food resources is critical for the WELF nexus, and this goes beyond resource access (Villamor et al., 2020). It includes the capacity to utilize these resources, and the dynamics and power relationships that affect the management of these resources (G. Villamor et al., 2020). Villamor et al. (2020) found that four aspects differentiate male and female perspectives with regards to WELF nexus resources and these differences include: (i) access to external actors; (ii) perceptions of target resources; (iii) gender specific productive roles; and (iv) decision-making with respect to target resource management and utilization, which may influence the dynamics and governance of the nexus. Overlooking these differences may make it difficult for the nexus approach to achieve gender equity while further aggravating the already burdening roles of women and children within households (Villamor et al., 2020). Resource inequality and access contribute to social instability and insecurity, therefore, incorporating gender perspectives into the nexus concept might help to identify specific local factors that may determine the degree of resource security and sustainable management (Villamor et al., 2020).

Climate change is having a negative impact and increasing pressure on WELF resources in SSA (Nhamo et al., 2018). All four sectors are highly vulnerable to climate change impacts and they also contribute heavily to that change through their greenhouse gas (GHG) emissions (Rasul and Sharma, 2016). Therefore climate change adaptation is intrinsically linked to these sectors (Rasul and Sharma, 2016). Water resources will be the hardest hit by climate change and these impacts are projected to increase in the future (Nhamo et al., 2018). The impacts include high temperatures, especially in the inland tropics, increased rainfall variability, increasing aridity, and increased frequency and intensity of droughts, floods, and extreme heat events (Serdeczny et al., 2017; Nhamo et al., 2018), which are affecting food production and energy generation. Sub-Saharan Africa is said to be the most vulnerable region to the impacts of global climate change because of its reliance on natural resources and agriculture, in particular, rain-fed agriculture, which is highly sensitive to weather and climate variables, and its low capacity to adapt, due to the high levels of relative poverty in the region (Kotir, 2011; Serdeczny et al., 2017). Thus, any negative effect of climate on the water cycle can threaten agriculture production, livelihoods and economy (Kotir, 2011; Nhamo et al., 2018). These challenges are exacerbated by population increase and industrial growth (Nhamo et al., 2018). A large proportion of SSA’s population depends on agriculture and as the risk to agricultural livelihoods increases, so will the rate of rural–urban migration, adding to the challenges of urbanization in the region (Serdeczny et al., 2017).

The impacts of climate change are not gender neutral nor are the adaptation strategies (Habtezion S., 2012; Sultana, 2018). Climate change-induced ecological and hydrological changes compound gender disparities in income, health, and education by exacerbating existing development challenges (Sultana, 2018). Men and women tend to have different coping and adaptive capacities and these translate to gender-differentiated vulnerabilities (Habtezion S., 2012). The productive and reproductive roles of women within the household make them increasingly vulnerable to climate change impacts, especially in rural areas as they are responsible for providing food for their households and procuring fuelwood for cooking and drinking water (Villamor et al., 2020). The gendered social roles and gender-based inequalities in access to assets are the primary reasons for the differences in adaptive capacities between men and women (Habtezion S., 2012). Moreover, legal and sociocultural barriers also affect women’s capacity to respond effectively to climatic risk (Habtezion S., 2012). Despite its gender neutrality, the WELF nexus presents an opportunity for a coordinated resource management (Nhamo et al., 2018) which will allow for a more equitable distribution of resources and the reduction of vulnerabilities, and the promotion of gender equality.

National and local policies relating to water, energy, land, food security/agriculture, and climate change within SSA need to acknowledge the importance of gender to the WELF nexus and climate change response. In order to advance climate-resilient strategies in the areas related to water, energy, land, and food, the incorporation of gender is crucial, especially for gender equality and operational effectiveness (Thuo et al., 2017). Gender-responsive planning for the WELF nexus will need to acknowledge and address power differentials among men and women within the sectors (Thuo et al., 2017). In mainstreaming gender into the nexus, the different roles of women and men need to be taken into account, and they need to be involved in policy and decision-making processes (Thuo et al., 2017; Sultana, 2018). It is also important to create outcomes that support both functional and strategic gender needs (Thuo et al., 2017). Sultana (2018) notes that while some programmes may address practical gender needs, they fail to address strategic gender needs and systemic gender inequalities, power structures, and exclusions. As a result, masculine bias remains in access to information, employment opportunities, decision-making processes, and institution building (Sultana, 2018).

Capacity needs to be developed for the integration of gender into integrated WELF planning and policies, and gender-responsive planning; funds need to be allocated; and there needs to be political will (Thuo et al., 2017). Care should be taken to not paint women and children as weak and vulnerable but they need to be viewed as actors who have agency and can make significant contributions to decision-making (Thuo et al., 2017). Moreover, it should be acknowledged that despite the increasing awareness on gender relations, cultural biases may affect any progress being made to address gender gaps in planning (Thuo et al., 2017). These biases may also affect the manner in which women participate, adding to barriers in planning and decision-making processes (Thuo et al., 2017).

Following the integration of gender into WELF Nexus and climate resilient planning, there needs to be monitoring and evaluation of progress. Clear indicators of what policy-makers are hoping to achieve need to be developed, as well as transparent processes of how these goals will be met (Thuo et al., 2017). Gendered knowledge about water, energy, land and food production can bolster adaptation programmes in different localities, however, these knowledge systems are often not engaged and individuals or groups do not fully participate in decision-making (Sultana, 2018). Gender inequality and the lack of involvement of differently located women in public decision also compromises the effectiveness of adaptation programmes (Sultana, 2018). The aim of these programmes is to reduce vulnerability to both climatic and non-climatic changes (Rasul and Sharma, 2016), and this is closely linked to achieving the sustainable use and management of water, energy, land and food resources, which are important for water, energy, and food security, as well as sustainable development (Rasul and Sharma, 2016).

Some projects have been launched in SSA to promote women’s access to-, and utilization of WELF resources. For example, in Benin, a pilot irrigation project was launched in 2007 by Stanford’s Woods Institute for the Environment, in partnership with Solar Electric Light Fund (SELF) (GWP SA, 2019). The project installed three solar-powered drip irrigation systems in two arid rural villages in Benin’s Kalalé district, Dunkassa and Bessassi (Eaton, 2012; GWP SA, 2019) to provide a cost-effective and environmentally friendly way to pump water for irrigation from nearby rivers and underground aquifers (UNFCCC, 2016). The project supplies local women’s cooperatives with water to grow fresh vegetables for consumption and sale, all year-round (Eaton, 2012; UNFCCC, 2016). These vegetables include tomatoes, okra, peppers, eggplants, amaranth, and carrots (GWP SA, 2019). The women in the co-operatives share the maintenance costs of the new irrigation technology (GWP SA, 2019). In addition to supplying water, the irrigation system freed women from the responsibility of having to fetch water to grow vegetables, especially during the 6-month dry season (GWP SA, 2019), allowing them to partake in educational and economic activities (Eaton, 2012). As a result, women are also empowered to become entrepreneurs and leaders in their communities (UNFCCC, 2016), while implementing solutions that promote food and nutrition, and climate change mitigation and adaptation. The initiative illustrates the role that renewable energy can play in creating new economic opportunities, while bringing water and food to poor communities, and it can be replicated, globally, especially in SSA (GWP SA, 2019; UNFCCC, 2016).

## Conclusion

Access to water, energy and food resources is highly gendered in sub-Saharan Africa, especially in rural areas, and women face numerous socio-cultural, economic, legal, and institutional barriers in accessing them, despite their productive and reproductive roles in the household. These resources are crucial for food security and human existence and how they are governed affects outcomes in terms of social equity, externalities, and socio-ecological resilience (Albrecht et al., 2018). The failure of women to access these resources makes them vulnerable, leaving them with a limited capacity to respond to the impacts of climate change, and a limited ability to harness opportunities arising from it. Although some countries in the region have made attempts to incorporate gender equality into their respective policies and eliminate gender discrimination, the implementation of these policies has been fraught with numerous challenges, including the micro-level dynamics within communities and households (Sultana, 2018), which add to the complexity of the challenges. Therefore, any efforts to address gender inequalities with regards to resource access need to address strategic gender needs and systemic gender inequalities, power structures, and exclusions (Sultana, 2018), while defeminizing resource inequality.

Given the interlinkages that exist between water, land, energy, and food, and the fact that access to these resources is gendered, gender needs to be mainstreamed into discussions about the WELF nexus. Adopting the nexus approach to resource management provides governments with an opportunity to create an enabling environment to improve equity in natural resource access; improve resources use efficiency; and abandon silo thinking within the different sectors (Ringler et al., 2013), while contributing climate change adaptation, and multiple goals of sustainable development. This can be achieved by the active involvement of both men and women in decision-making about these resources, and climate change response is crucial to the success to any plans or programmes that are developed and implemented to address these challenges. Furthermore, gender relations need to be transformed and men need to be receptive to the change, and view women as partners. Transforming gender relations by allowing women to have the same access to resources as men and empowering them as decision-makers will have multiplier effects on production, productivity, efficiency, inclusive growth and poverty reduction in the region (Farnworth et al., 2013; Ben-Ari, 2014; Sakho-Jimbira and Hathie, 2020). It is also important to implement effective monitoring and reporting mechanisms in order to track progress systematically (Ghebru, 2019).
